# Integrated stress response activator halofuginone protects mice from diabetes-like phenotypes

**DOI:** 10.1083/jcb.202405175

**Published:** 2024-08-16

**Authors:** Shashank Rai, Maria Szaruga, Aleksandra P. Pitera, Anne Bertolotti

**Affiliations:** 1 MRC Laboratory of Molecular Biology, Cambridge, UK

## Abstract

The integrated stress response (ISR) is a vital signaling pathway initiated by four kinases, PERK, GCN2, HRI and PKR, that ensure cellular resilience and protect cells from challenges. Here, we investigated whether increasing ISR signaling could rescue diabetes-like phenotypes in a mouse model of diet-induced obesity (DIO). We show that the orally available and clinically approved GCN2 activator halofuginone (HF) can activate the ISR in mouse tissues. We found that daily oral administration of HF increases glucose tolerance whilst reducing weight gain, insulin resistance, and serum insulin in DIO mice. Conversely, the ISR inhibitor GSK2656157, used at low doses to optimize its selectivity, aggravates glucose intolerance in DIO mice. Whilst loss of function mutations in mice and humans have revealed that PERK is the essential ISR kinase that protects from diabetes, our work demonstrates the therapeutic value of increasing ISR signaling by activating the related kinase GCN2 to reduce diabetes phenotypes in a DIO mouse model.

## Teaser

This work shows that activation of the integrated stress response (ISR) with orally available and clinically approved GCN2 activator halofuginone reduces diabetes-like phenotypes in a mouse model of diet-induced obesity. Conversely, ISR inhibition aggravates glucose intolerance in obese mice. This demonstrates the therapeutic value of increasing ISR signaling in diabetes.

## Introduction

The integrated stress response (ISR) is a vital homeostatic pathway activated by four kinases, PERK, GCN2, PKR, and HRI that phosphorylate the alpha subunit of the eukaryotic translation initiation factor 2 (eIF2α) on serine 51 to slow down translation initiation and induce transcriptional reprograming, thereby allowing cells to survive changes in their environment (Sonenberg and Hinnebusch, 2009; Wek, 2018). PERK is activated by misfolded proteins in the endoplasmic reticulum (ER) to decrease protein synthesis thus preventing overwhelming the folding capacity of the ER. GCN2 is activated by amino acid shortage, PKR by double-stranded RNA during viral infections, and HRI by heme deficiency (Sonenberg and Hinnebusch, 2009; Wek, 2018). Two eIF2α phosphatases reverse the activity of the four eIF2α kinases. They are composed of a common catalytic subunit, protein phosphatase 1 (PP1), bound to one of the two specific substrate receptor subunits: the stress-inducible PPP1R15A (R15A) or the constitutive PPP1R15B (R15B) (Bertolotti, 2018). The antagonistic actions of the four eIF2α kinases and the two phosphatases adjust the levels of phosphorylation of eIF2α to the cellular needs and conditions. Loss of function mutations in PERK cause a rare autosomal recessive disease characterized by neonatal or early-onset diabetes (Delépine et al., 2000), and mice lacking a functional PERK or a phosphorylation site on its substrate eIF2α also die shortly after birth because they fail to regulate glucose homeostasis due to β-cell loss (Harding et al., 2001; Scheuner et al., 2001).

PERK is common to both the ISR and the ER unfolded protein response (ER-UPR). Whilst the ER-UPR functions in all cells, it is considered to be particularly important to pancreatic β-cells due to the high biosynthetic burden that the production of insulin poses on their ER. Insulin is synthesized as single-chain polypeptides, which is demanding on the ER of the pancreatic β-cells due to both the amounts produced (insulin representing >50% of the proteins produced by pancreatic β-cells) and its complex maturation (processing and disulfide bond formation) (Liu et al., 2021). The importance of the ER-UPR in diabetes has been abundantly studied with evidence that ER-UPR markers are elevated in mouse models of diabetes as well as in human patients (Morikawa and Urano, 2022). Rare genetic disorders with mutations in genes affecting ER homeostasis reinforce the mechanistic links between ER dysfunction and diabetes (Morikawa and Urano, 2022). The benefit of pharmacological manipulation of the ER-UPR, consisting of pharmacological chaperones and drugs affecting ER calcium levels, has also been evaluated in animal models and in early clinical trials (Liu et al., 2021).

Although pharmacological manipulation of the ISR is gaining a lot of attention in both academia and pharmaceutical companies, its therapeutic potential for the maintenance of glucose homeostasis is understudied. Based on direct and converging evidence from mice to humans establishing that the ISR is vital for β-cells survival, we anticipated that a suitable manipulation of the ISR may provide a mechanism-based therapeutic approach to reduce glucose intolerance in DIO. The ISR has emerged as a prime target for new pharmacological manipulations, with compounds that either prolong or inhibit eIF2α phosphorylation or its downstream signaling (Costa-Mattioli and Walter, 2020; Luh and Bertolotti, 2020; Tian et al., 2021). The therapeutic benefit of increasing or decreasing ISR signaling has been reported in various disease models (Costa-Mattioli and Walter, 2020; Luh and Bertolotti, 2020). Here, we took an unprecedented approach consisting of comparing side-by-side pharmacological activation and inhibition of ISR in DIO mice to assess the potential of targeting this pathway in this condition.

## Results

### Oral administration of HF induces the ISR in mouse tissues

**Figure 1.**
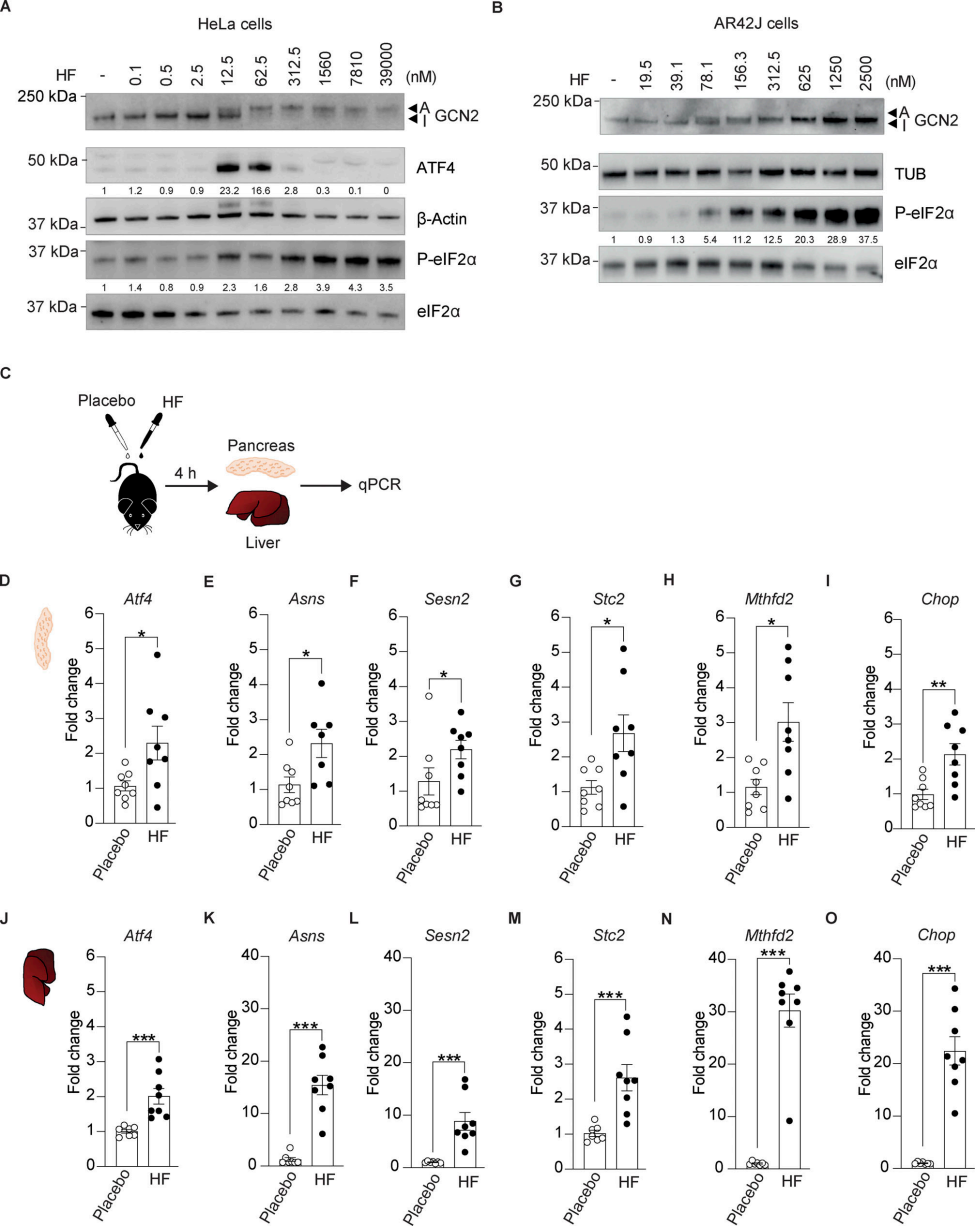
**Oral administration of halofuginone induces the ISR in mouse tissues. (A)** Immunoblots of indicated proteins from lysates of HeLa cells untreated or treated with increasing concentrations of HF. Representative experiment from *n* = 3, biological replicates. ATF4 and P-eIF2α levels have been quantified using ImageStudio Lite software and normalized to untreated. **(B)** Immunoblots of indicated proteins from lysates of AR42J cells untreated or treated with increasing concentrations of HF. A representative experiment from *n* = 3, biological replicates. P-eIF2α levels have been quantified using ImageStudio Lite software and normalized to untreated. Note that the ATF4 antibody used in this study did not recognize the rat protein from AR42J cells. **(C)** Schematic of treatments of 6–7-wk-old C57BL6/J males with either placebo or 0.5 mg/kg HF. Pancreas were harvested 4 h after dosing for RNA extraction and gene expression analysis. **(D–O)** Relative abundance of the indicated mRNAs detected by qPCR in lysates of pancreas (D–I) and liver (J–O) from mice treated with placebo or 0.5 mg/kg HF for 4 h. Data are shown as mean ± SEM (*n* = 7 or 8). *P < 0.05, **P < 0.01 ***P < 0.001 as determined by two-tailed Mann-Whitney test. Source data are available for this figure: SourceData F1.

Aiming to test the potential of ISR activation in DIO mice, we chose halofuginone (HF), a potent, orally available ISR inducer that has progressed to phase 2 clinical trials for various indications (https://clinicaltrials.gov). HF was designed as a derivative of febrifugine, a natural product that has been used for centuries in Chinese medicine to treat malaria (Pines and Spector, 2015). It has been widely utilized in veterinary medicine for more than two decades to treat parasites in poultry and cattle (Daugschies et al., 1998). Thus, primary safety is not a concern for this compound, as it is broadly used in animals and has been used in phase 2 clinical trials in humans. HF binds and inhibits the glutamyl-prolyl-tRNA synthetase with low nanomolar potency and as a result induces the ISR by activating GCN2 (Keller et al., 2012; Sundrud et al., 2009; Pitera et al., 2022). As previously shown, HF activated GCN2 in cells in a dose-dependent manner, and this resulted in a shift in its mobility on polyacrylamide gel electrophoresis (Fig. 1 A). As expected, this resulted in a parallel increase in the phosphorylation of eIF2α (Fig. 1 A). However, whilst the ISR target ATF4, downstream of eIF2α phosphorylation, increased upon treatment with 12.5 and 62.5 nM of HF, this was no longer observed with higher concentrations (Fig. 1 A). The blunted ISR at high concentration of HF occurs because saturating concentrations of HF completely inhibit the glutamyl-prolyl-tRNA synthetase, thereby depleting the pool of prolyl-tRNA, and as a result, causing translation pausing on proline codons (Misra et al., 2021; Pitera et al., 2022). Thus, HF is a dose-dependent ISR inducer at low to moderate concentrations but blocks translation elongation at saturating concentrations (Pitera et al., 2022). In the nanomolar and low micromolar range, HF also activated GCN2 and induced the ISR in pancreatic cells (Fig. 1 B). We previously reported that the diverse measurable activities of HF are mediated by an on-target mechanism consisting of inhibiting the glutamyl-prolyl-tRNA synthetase resulting in GCN2 activation (Pitera et al., 2022).

The ISR-activating properties of HF at low concentrations, its safety and efficacy profile attested by decades of use in veterinary practice, and its suitability for phase 2 use in human clinical trials distinguish HF as a best-in-class compound to explore the potential benefit of increasing ISR signaling to mitigate diabetes-related phenotypes in vivo. For mouse studies, we used a commercially available formulation of HF (see Materials and methods) widely and safely used for oral treatment of cattle against parasites (Pines and Spector, 2015).

To select adequate ISR-inducing concentrations for in vivo studies in mice, we designed our dosing based on the previously published pharmacokinetics and tissue distribution of HF after oral gavage in rats (Stecklair et al., 2001). We treated a cohort of 16 mice of 6–7 wk of age with a single oral dose of 0.5 mg/kg HF or an equal volume of placebo and used qPCR to monitor ISR activation in tissues collected 4 h after dosing (Fig. 1 C). We first examined ISR induction in the pancreas as this had not been assessed before. A single oral dose of 0.5 mg/kg HF increased the expression of the various ISR targets *Atf4*, *Asns*, *Chop*, *Sesn2*, *Stc2*, and *Mthfd2* by two- to threefold (Fig. 1, D–I). Broad-tissue distribution of HF has been previously demonstrated (Stecklair et al., 2001). We next examined ISR gene expression in the liver to assess the effect of the treatment in another organ. Induction of diverse ISR-target genes was robust in the liver (Fig. 1, J–O). These studies demonstrate that oral administration of HF induces ISR in mouse tissues.

### Oral administration of HF improves glucose tolerance in DIO mice

**Figure 2.**
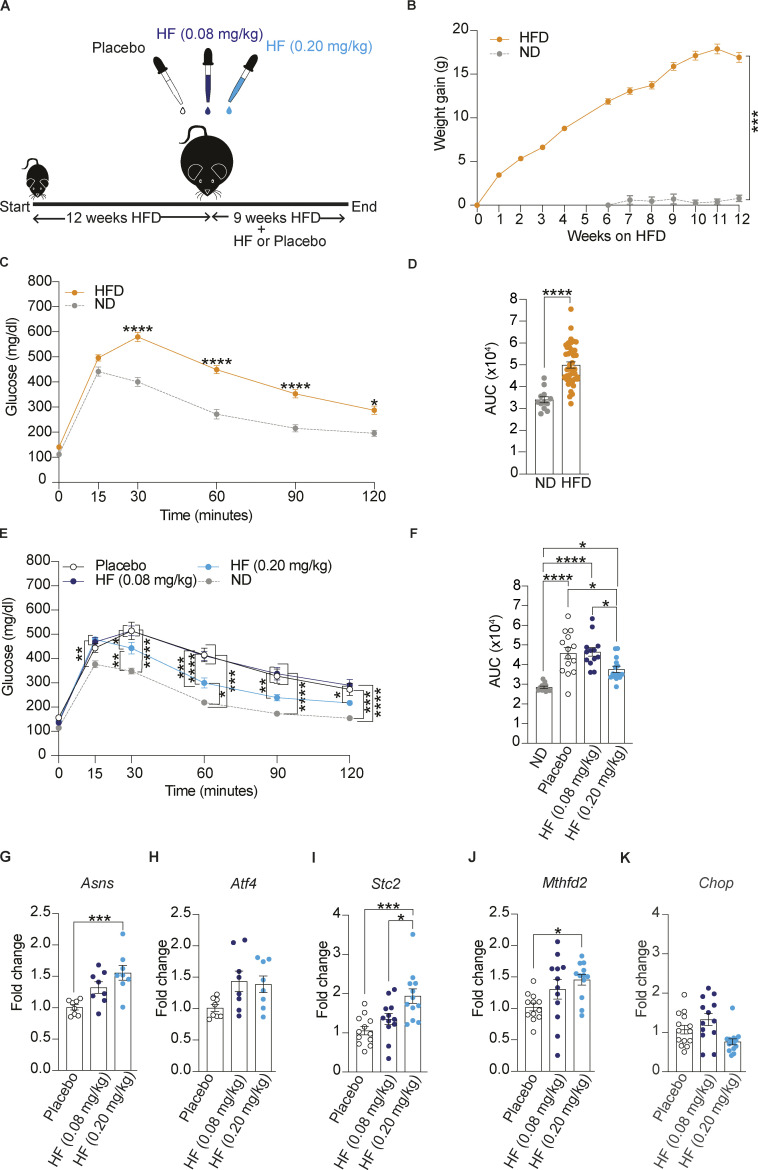
**Oral administration of halofuginone improves glucose tolerance in DIO mice. (A)** Schematic of experimental design. 6–7-wk old C57BL6/J males were fed on a high-fat diet (HFD) (60% kCal) for 12 wk as a single cohort before daily oral treatment with a placebo or two different concentrations of HF for 9 wk with continued HFD. **(B)** Weight gain of mice fed on normal diet (ND, 12 mice) or HFD (42 mice) for 12 wk. **(C and D)** Glucose tolerance test in DIO and ND-fed mice at the end of 12 wk of HFD. **(D)** Area under the curve (AUC). The 42 DIO mice group was divided into 3 groups of 14 mice each for treatment with HF (2 doses) or placebo after 12 wk on HFD. **(E and F)** Glucose tolerance test in DIO mice after 9 wk of placebo or HF (0.08 mg/kg) or HF (0.20 mg/kg) treatment with continued HFD. **(F)** Glucose tolerance represented as AUC. **(G–K)** Relative abundance of the indicated mRNAs detected by qPCR in lysates of the pancreas from mice treated with placebo or 0.08 or 0.20 mg/kg HF for 9 wk. Data are shown as mean ± SEM (*n* = 8 or 12–14). *P < 0.05, **P < 0.01, ***P < 0.001, and ****P < 0.0001 as determined by two-tailed unpaired *t* test (B and D), two-way ANOVA (C and E) and ordinary one-way ANOVA (F–K). Data distribution was assumed to be normal.

**Figure S1.**
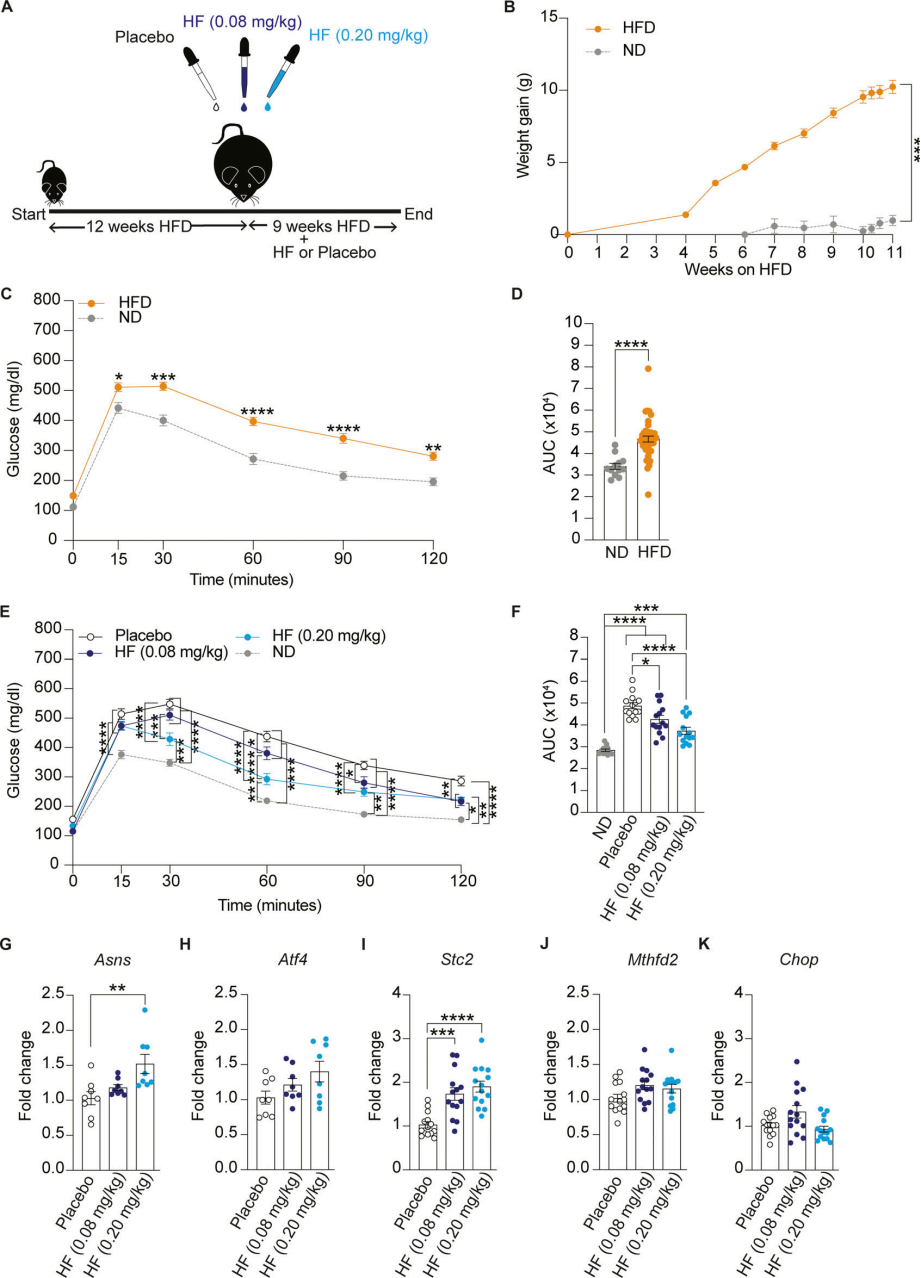
**Oral administration of halofuginone improves glucose tolerance in DIO mice.** Replicate of Fig. 2 with a separate DIO mouse cohort. **(A)** Schematic of experimental design. 6–7 wk old C57BL6/J males were fed on high-fat diet (HFD) (60% kCal) for 12 wk as a single cohort before daily oral treatment with a placebo or two different concentrations of HF for 9 wk with a continued HFD. **(B)** Weight gain of mice fed on a normal diet (ND, 12 mice) or HFD (42 mice) for 12 wk. **(C and D)** Glucose tolerance test in DIO and ND-fed mice at the end of 12 wk of HFD. **(D)** AUC of glucose tolerance test. The 42 DIO mice group was divided into three groups of 14 mice each for treatment with HF (2 doses) or placebo after 12 wk on HFD. **(E and F)** Glucose tolerance test in DIO mice after 9 wk of placebo or HF (0.08 mg/kg) or HF (0.20 mg/kg) treatment with continued HFD. **(F)** Glucose tolerance test is represented as AUC. The ND cohort shown here is the same as Fig. 2 and is included for reference. **(G–K)** Relative abundance of the indicated mRNAs detected by qPCR in lysates of pancreas from mice treated with placebo or 0.08 or 0.20 mg/kg HF for 9 wk. Data are shown as mean ± SEM (*n* = 14 or 12). *P < 0.05, **P < 0.01, ***P < 0.001, and ****P < 0.0001 as determined by two-tailed unpaired *t* test (B and D), two-way ANOVA (C and E), and ordinary one-way ANOVA (F–K). Data distribution was assumed to be normal.

To assess the potential therapeutic effect of inducing ISR in diabetes, we set out to test the effect of HF in diet-induced obese (DIO) mice after the mice had already developed disease phenotypes. Thus, we generated a cohort of DIO mice by feeding 42 C57BL/6J male mice with a high-fat diet (HFD; 60% fat in kcal) for 12 wk (Fig. 2 A). As expected, HFD-fed mice became obese (Fig. 2 B) and glucose-intolerant (Fig. 2, C and D). These DIO mice were then separated into three groups of 14 mice each and given placebo or HF daily by oral gavage using 0.2 and 0.08 mg/kg doses for the chronic treatments whilst continuing feeding them HFD. To assess the effect of HF, glucose tolerance was monitored after 9 wk of treatment. We found that an oral daily dose of 0.2 mg/kg HF caused a marked improvement in glucose tolerance in DIO mice (Fig. 2, E and F). To test the robustness of these findings, we repeated these experiments with a separate cohort of DIO mice (Fig. S1). We found that both 0.08 and 0.2 mg/kg HF improved glucose tolerance in this cohort (Fig. S1, E and F). Thus, activation of the ISR with the GCN2 activator HF significantly improves glucose tolerance in HFD-fed mice.

**Figure S2.**
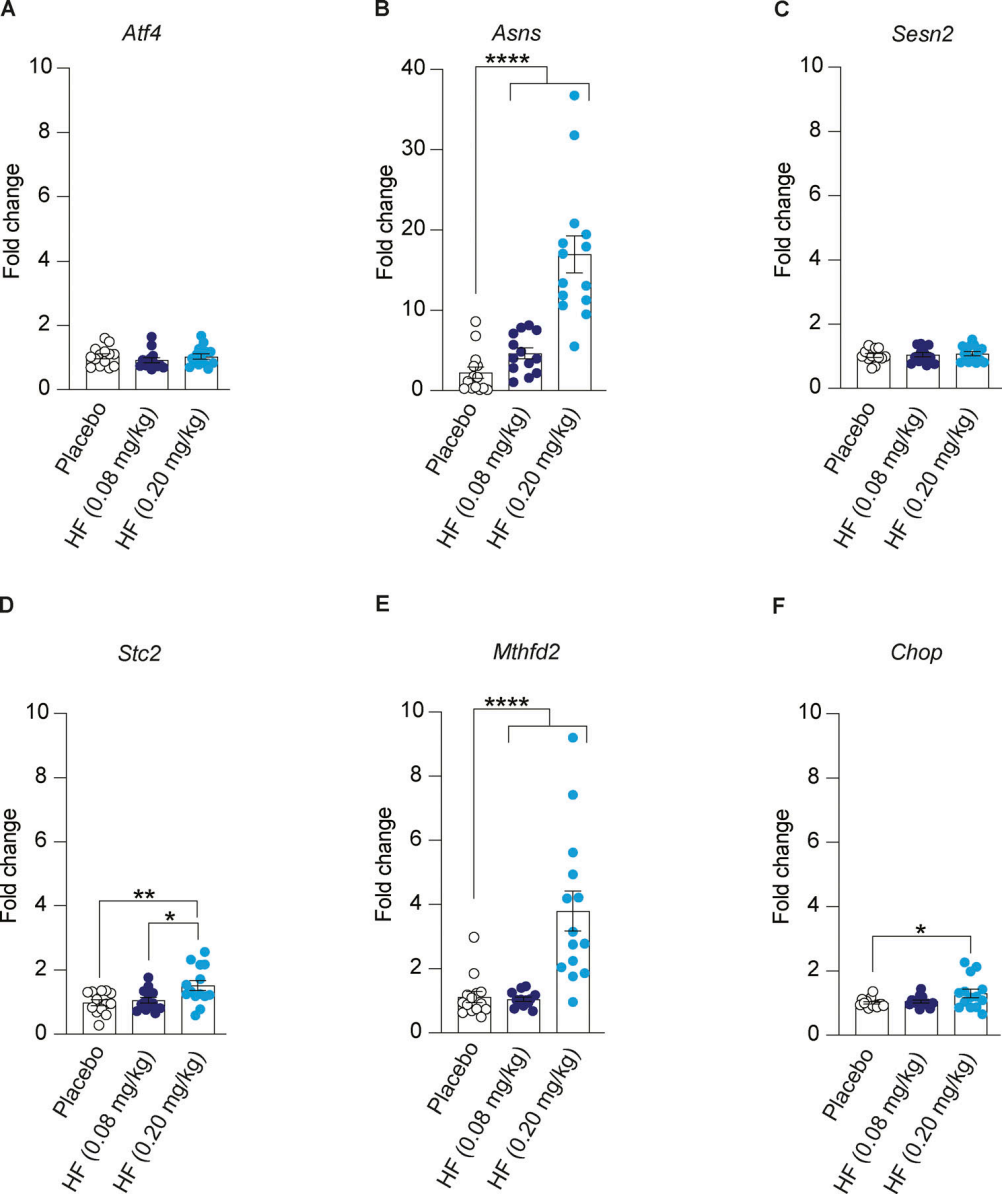
**Oral administration of halofuginone induces ISR in liver of DIO mice. (A–F)** Relative abundance of the indicated mRNAs detected by qPCR in lysates of liver from mice treated with placebo or 0.08 mg/kg or 0.20 mg/kg HF for 9 weeks. Data are shown as mean ± SEM (*n* = 14 or 12). *P < 0.05, **P < 0.01, and ****P < 0.0001 as determined by ordinary one-way ANOVA. Data distribution was assumed to be normal.

Seeking molecular evidence for this interpretation, we monitored the expression of ISR target genes in tissues collected from the DIO mice after chronic treatment with HF. We found that expression of ISR targets *Asns*, *Stc2*, and *Mthfd2* was significantly increased in the pancreas and the liver of DIO mice treated with 0.2 mg/kg HF (Fig. 2, G, I, and J; and Fig. S2, B, D, and E). A non-significant increase of *Asns* was observed with 0.08 mg/kg in both the pancreas and liver (Fig. 2 G and Fig. S2 B). Similar findings were observed in the second, independent DIO mouse cohort treated with 0.08 and 0.2 mg/kg HF (Fig. S1, G and I). This confirms that, at the doses used, HF activated the ISR in the pancreas and liver of DIO mice.

### Oral administration of HF reduces insulin resistance and weight gain in DIO mice

**Figure 3.**
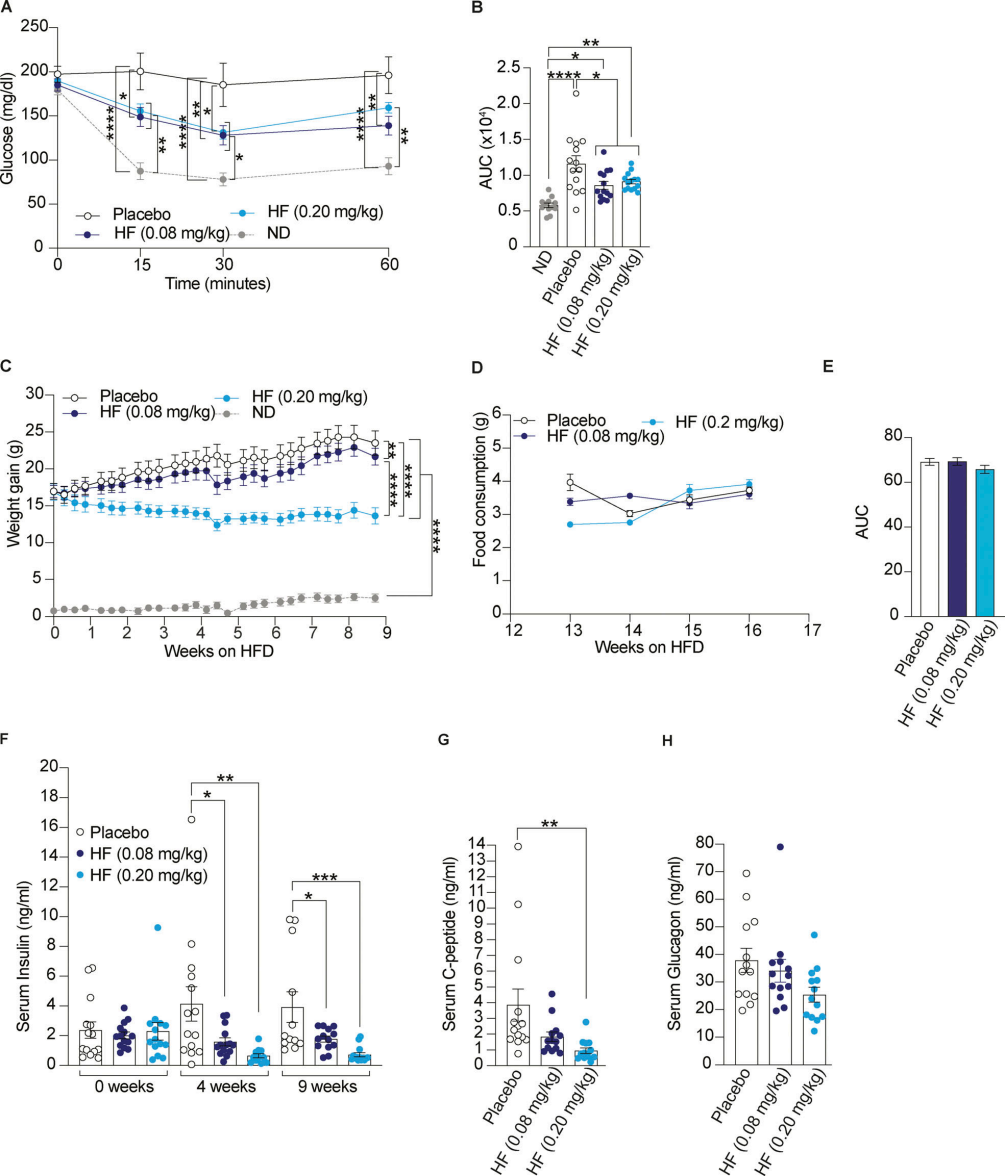
**Oral administration of halofuginone reduces insulin resistance, weight gain and serum insulin levels in DIO mice. (A and B)** Insulin tolerance test in DIO mice treated for 5 wk with either placebo or HF (0.08 or 0.20 mg/kg) and ND-fed mice. Mice were food-deprived for 5 h before receiving a single intraperitoneal injection of insulin (0.75 U/kg). **(B)** Insulin tolerance test presented as AUC plot. **(C)** Weight gain by DIO mice treated for 9 wk with either placebo or HF at indicated doses with continued HFD and ND-fed mice. **(D)** Food consumption comparison plot for HFD-fed mice treated with either placebo or HF (0.08 mg/kg) or HF (0.20 mg/kg). **(E)** AUC of food consumption plot. **(F–H)** Serum levels of insulin, (G) C-peptide, and (H) glucagon in DIO mice treated with either placebo or 0.08 or 0.20 mg/kg of HF. Data are shown as mean ± SEM (*n* = 12–14). *P < 0.05, **P < 0.01, ***P < 0.001, and ****P < 0.0001 as determined by two-way ANOVA (A), ordinary one-way ANOVA (B, C, E, and F–H). Data distribution was assumed to be normal.

**Figure S3.**
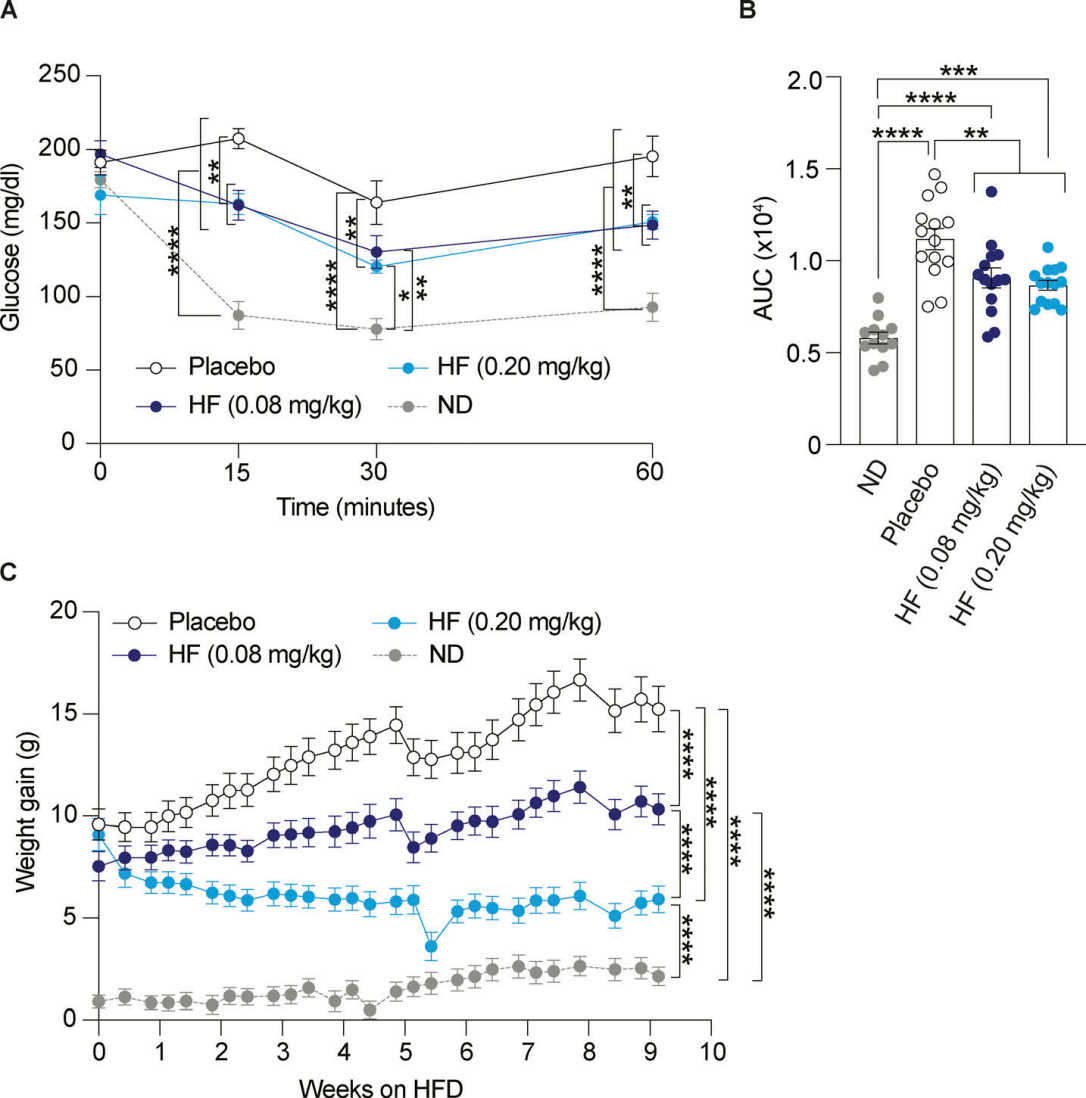
**Oral administration of halofuginone reduces insulin resistance and weight gain in DIO mice.** Replicate of Fig. 3 with a separate DIO mouse cohort. **(A and B)** Insulin tolerance test in DIO mice treated for 5 wk with either placebo or HF (0.08 or 0.20 mg/kg) and ND-fed mice. Mice were food-deprived for 5 h before receiving a single intraperitoneal injection of insulin (0.75 U/kg). **(B)** Insulin tolerance test presented as AUC plot. **(C)** Weight gain in DIO mice treated for 9 wk with either placebo or HF at indicated doses with continued HFD and ND-fed mice. The ND cohort shown here is the same as Figs. 2 and 3 and is included for reference. Data are shown as mean ± SEM (*n* = 14 or 12). *P < 0.05, **P < 0.01, ***P < 0.001, and ****P < 0.0001 as determined by two-way ANOVA (A) and ordinary one-way ANOVA for B and C. Data distribution was assumed to be normal.

DIO is associated with insulin resistance and hyperglycemia. To examine if HF affected insulin resistance, we subjected the same cohort of mice to insulin tolerance tests and monitored glucose clearance over time. We found that 5 wk of HF treatment improved glucose clearance after injection of insulin indicating that HF reduced insulin resistance (Fig. 3, A and B). In parallel, we continued to monitor weight gain by weighing mice thrice weekly for the entire period of study. Mice treated with 0.20 mg/kg HF stopped gaining weight soon after treatment started compared with mice treated with placebo or the lower dose of HF (Fig. 3 C). During the 9 wk of the treatment, the weight of mice dosed with 0.20 mg/kg HF remained stable despite the HFD (Fig. 3 C). Food consumption was monitored during the treatment (Fig. 3 D) and overall, no statistically significant differences were observed between placebo or HF-treated mice (Fig. 3 E). To assess the reproducibility of these findings, we repeated these analyses in a separate experimental cohort of DIO mice. We found that both 0.08 and 0.2 mg/kg HF reduced insulin resistance and weight gain in DIO mice (Fig. S3). This shows that HF reduces insulin resistance and prevents weight gain in DIO mice.

### HF reduces insulin levels in diet-induced diabetic mice

The decreased glucose tolerance on HFD causes increasing demands on insulin synthesis to try to maintain normoglycemia and with the associated insulin resistance in tissues, pancreatic β- cells respond by producing more insulin, a vicious cycle leading to hyperinsulinemia. Thus, we next tested if HF treatment can affect serum insulin levels in DIO mice. Blood serum was collected from all mice at 0, 4, and 9 wk of HF or placebo treatment, and insulin levels were measured by ELISA. We found that insulin levels in the serum of mice treated with 0.2 or 0.08 mg/kg HF were significantly lower than in placebo-treated mice after 4 and 9 wk of treatment, revealing that HF reduced hyperinsulinemia (Fig. 3 F). We also found that the levels of C-peptide significantly decreased upon treatment with HF, further confirming that HF reduced insulin levels in DIO mice (Fig. 3 G). In contrast, the differences in serum levels of glucagon were statistically insignificant upon HF treatment (Fig. 3 H). This shows that HF treatment decreases insulin levels in DIO mice.

### Selecting dosing for PERK inhibition with GSK’157 in vivo

Diverse experimental readouts demonstrate that ISR activation with HF reduces glucose intolerance and obesity in mice fed with HFD, a medically relevant paradigm. To consolidate these findings, we set out to exert the opposite pharmacological manipulation of the ISR and assessed the consequences of inhibiting PERK in DIO mice. GSK2656157 (GSK’157) is a nanomolar PERK inhibitor (Atkins et al., 2013). In addition to its biochemical potency in vitro, in cells, and in vivo, the inhibitor was reported to be >100-fold selective over >300 kinases (Atkins et al., 2013). GSK’157 is orally available and shows a dose- and time-dependent pharmacokinetic and pharmacodynamic activity in mouse pancreas (Atkins et al., 2013). The efficacy of the inhibitor was first established in tumor xenografts, upon bi-daily treatment with the inhibitor at 50 or 150 mg/kg (Atkins et al., 2013). This inhibitor has now been used in various in vivo studies using similar dosing, and efficacy was reported in a range of disease models, from cancer to neurodegeneration (Rozpedek-Kaminska et al., 2020; Tian et al., 2021). The dosing with the first study with GSK’157 was designed to provide proof of concept for the ability of GSK’157 to inhibit its target in vivo and therefore used excessive doses to ensure saturated binding of PERK and its full inhibition (Atkins et al., 2013). Based on the pharmacokinetic profile of GSK’157, dosing of 50 mg/kg results in 24 μM of the inhibitor in the pancreas, a dose equivalent to ∼2,500 times the EC_50_ in cells (Atkins et al., 2013). At such high concentrations, the inhibitor is no longer selective (Atkins et al., 2013; Rojas-Rivera et al., 2017) and diverse ISR markers such as *Asns* were elevated despite PERK being inhibited in the pancreas as well as in human tumor xenografts (Atkins et al., 2013). This was explained by our findings revealing that micromolar concentrations of the PERK inhibitor activate GCN2 and the ISR rather than inhibiting it (Szaruga et al., 2023). The development of PERK inhibitors was paused due to pancreatic damage observed when treating mice, rats, and dogs with high doses (50–150 mg/kg) of GSK’157 (Atkins et al., 2013). It is not known whether the pancreatic damage is dose-dependent, but it is reasonable to anticipate that toxicity of the inhibitor might become manageable with reduced dosing. Thus, the recent comprehensive knowledge of dose-response and relative selectivity of GSK’157 for PERK when used at submicromolar concentrations (Szaruga et al., 2023) opens the possibility to use the inhibitor in the submicromolar range for selective inhibition of the ISR.

**Figure 4.**
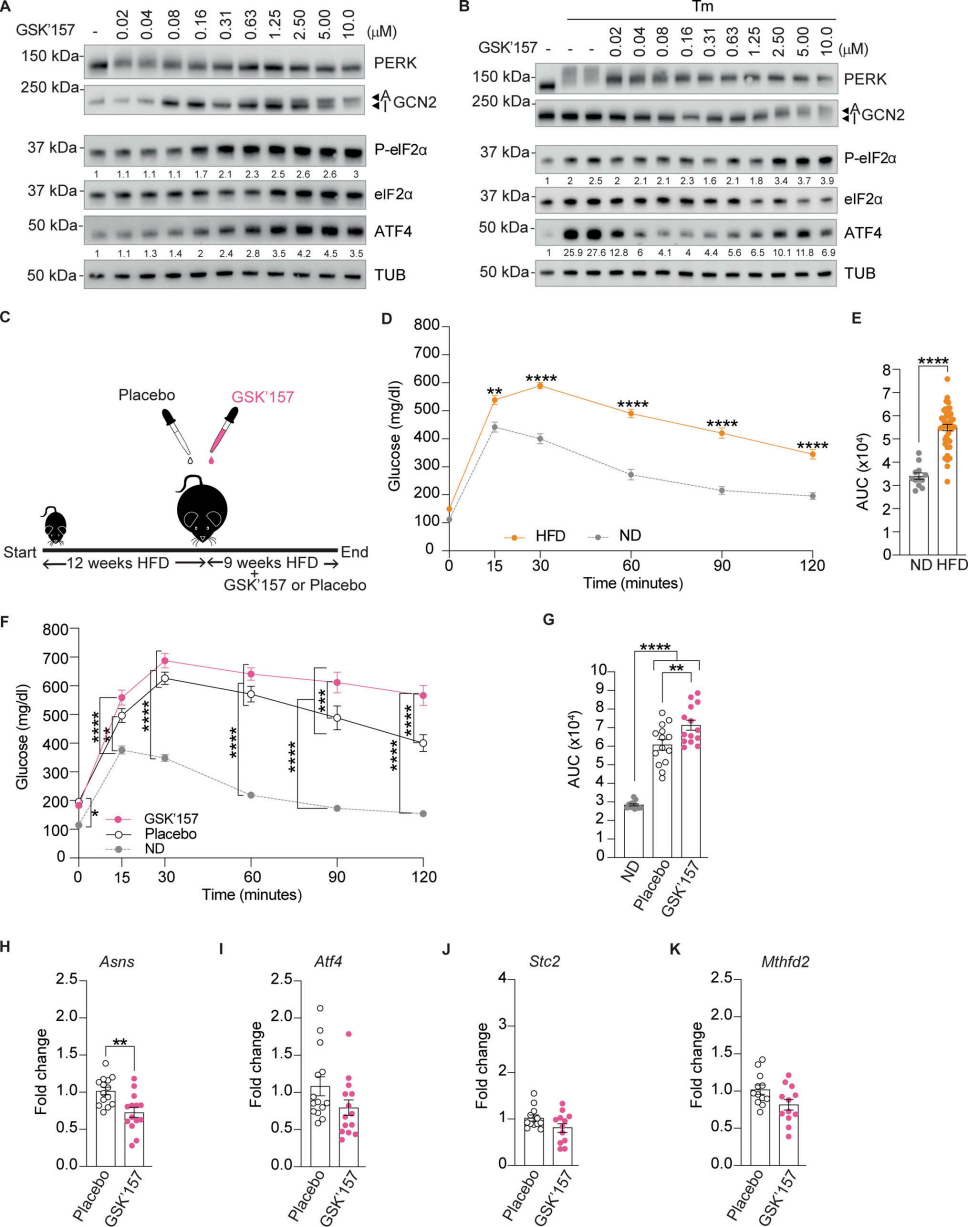
**Low dose of PERK inhibitor GSK’157 aggravates glucose intolerance in DIO mice. (A)** Immunoblots of indicated proteins from lysates of HeLa cells untreated or treated with increasing concentrations of GSK’157. Representative image from *n* = 3, biological replicates. ATF4 and P-eIF2α levels have been quantified using ImageStudio Lite software and normalized to untreated. **(B)** Immunoblots of indicated proteins from lysates of HeLa cells untreated or treated with Tunicamycin and increasing concentrations of GSK’157. Representative image from *n* = 3, biological replicates. ATF4 and P-eIF2α levels have been quantified using ImageStudio Lite software and normalized to untreated. **(C)** Schematic of treatments of 6–7-wk old C57BL6/J males with either placebo or 0.1 mg/kg GSK’157. **(D and E)** Glucose tolerance test of DIO mice at the end of 12 wk of HFD. **(E)** AUC of glucose tolerance test. The 28 DIO mice group was divided in two groups of 14 mice each for treatment with GSK’157 (0.1 mg/kg) or placebo after 12 wk on HFD. **(F and G)** Glucose tolerance test in DIO mice after 9 wk of placebo or GSK’157 (0.1 mg/kg) treatment with a continued HFD. **(G)** Glucose tolerance test is represented as AUC. The ND cohort shown here is the same as Fig. 2 and is included for reference. **(H–K)** Relative abundance of the indicated mRNAs detected by qPCR in lysates of the pancreas from mice treated with placebo or 0.1 mg/kg GSK’157 for 9 wk. Data are shown as mean ± SEM (*n* = 14 or 12). *P < 0.05, **P < 0.01, ***P < 0.001, and ****P < 0.0001 as determined by two-way ANOVA for D and F, ordinary one-way ANOVA (G), two-tailed unpaired *t* test (E), and two-tailed Mann-Whitney test (H–K). Data distribution was assumed to be normal. Source data are available for this figure: SourceData F4.

As previously reported (Szaruga et al., 2023), in cells, GSK’157 activated the ISR at concentrations ranging from 80 nM to 10 μM (Fig. 4 A). Here, we aimed at using GSK’157 at ISR-inhibiting concentrations, without the confounding effect of GCN2 activation. Thus, we performed a dose-response of GSK’157 in Tunicamycin-treated cells to assess PERK and ISR inhibition (Fig. 4 B). As expected (Atkins et al., 2013), we found that GSK’157 inhibited Tunicamycin-activated PERK over a broad range of concentrations and resulted in ISR inhibition in the low nanomolar range of the inhibitor (Fig. 4 B). Aiming to inhibit the ISR in vivo, we used 0.1 mg/kg GSK’157, a dose selected to ensure low nanomolar exposure of the pancreas to the compound, based on the pharmacokinetic and pharmacodynamic properties of the compound (Atkins et al., 2013).

### Low dose of PERK inhibitor GSK’157 aggravates glucose intolerance in DIO mice

As before, we generated a DIO cohort feeding 28 C57BL/6J male mice on HFD for 12 wk (Fig. 4 C). As expected, HFD-fed mice became glucose-intolerant (Fig. 4, D and E). This DIO cohort was then separated into 2 groups and given placebo or 0.1 mg/kg GSK’157 by oral gavage daily whilst remaining on HFD. Glucose tolerance was monitored after 9 wk of treatment. We found that an oral daily dose of 0.1 mg/kg GSK’157 aggravated glucose intolerance in DIO mice (Fig. 4, F and G). Thus, inhibition of the ISR with a daily dose of 0.1 mg/kg GSK’157 significantly aggravated HFD-induced glucose intolerance in DOI mice. We found that DIO mice treated with 0.1 mg/kg of GSK’157 showed a significant decrease in *Asns* expression in their pancreas (Fig. 4 H). This confirms that, at the low dose used, GSK’157 inhibited the ISR in the pancreas of DIO mice.

**Figure 5.**
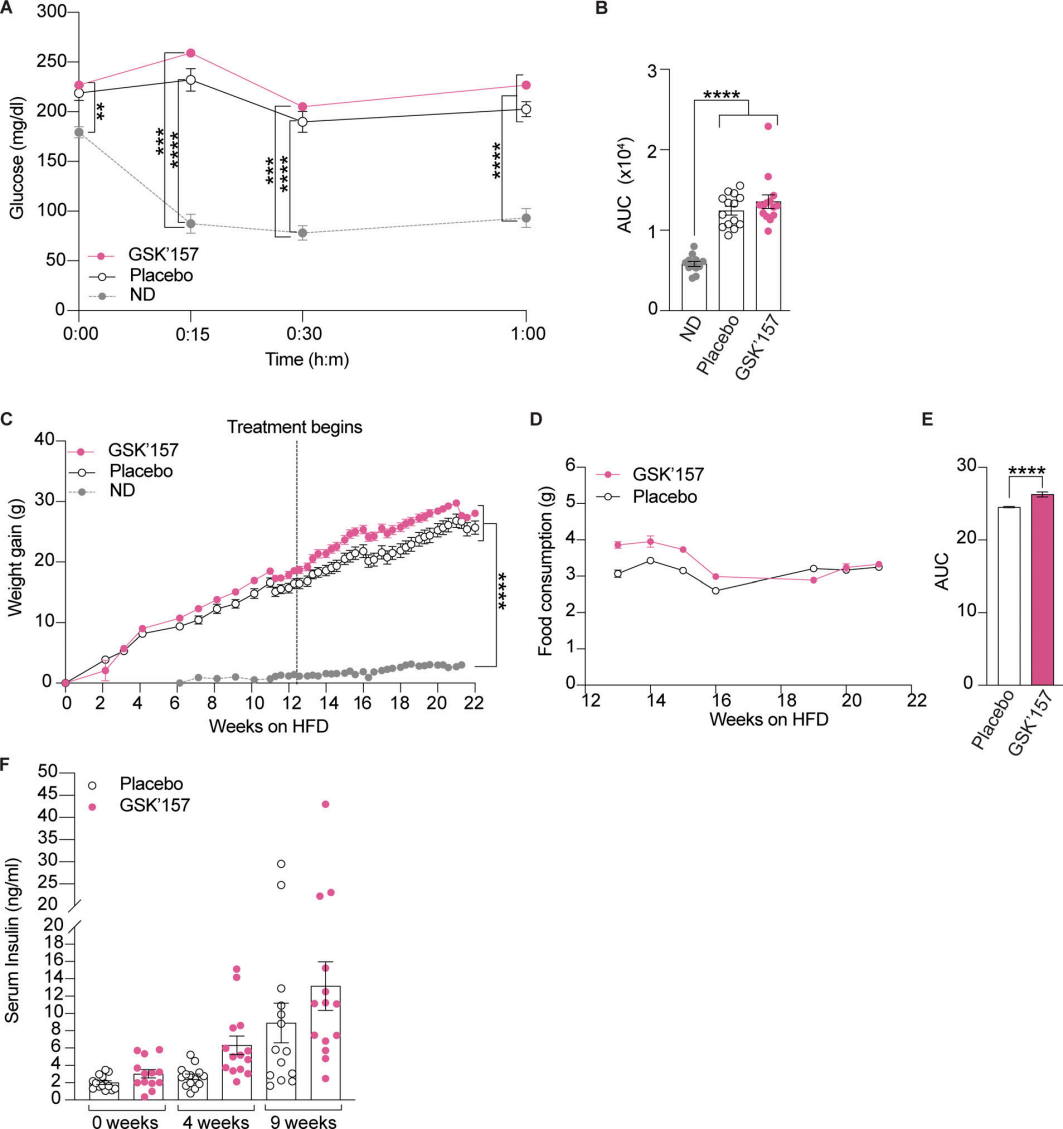
**Effect of low dose of GSK’157 on insulin resistance, weight gain, and serum insulin levels. (A and B)** Insulin tolerance test in DIO mice treated for 5 wk with either placebo or GSK’157 (0.1 mg/kg). Mice were food-deprived for 5 h before receiving a single intraperitoneal injection of insulin (0.75 U/kg). **(B)** Insulin tolerance is presented as an AUC plot. **(C)** Weight gain by DIO mice treated for 9 wk with either placebo or GSK’157 (0.1 mg/kg) with a continued HFD. **(D and E)** Food consumption comparison plot for HFD-fed mice treated with either placebo or GSK’157 (0.1 mg/kg). **(E)** AUC of food consumption plot. **(F)** Serum levels of insulin in DIO mice treated with either placebo or GSK’157 (0.1 mg/kg). The ND cohort shown here is the same as Figs. 2 and 3 and is included for reference. Data are shown as mean ± SEM (*n* = 14 or 12). **P < 0.01, ***P < 0.001, and ****P < 0.0001 as determined by two-way ANOVA (A), ordinary one-way ANOVA (B, C, and F), and two-tailed unpaired *t* test (E). Data distribution was assumed to be normal.

We next assessed insulin resistance 5 wk after daily oral treatment of GSK’157 and observed no significant differences relative to the placebo group (Fig. 5, A and B). Weight monitoring over the 9 wk of the treatment showed that GSK’157 tended to increase weight gain, although the differences were not statistically significant (Fig. 5 C). Food intake was monitored during the treatment and mice with GSK’157 treatment consumed significantly more amount of HF food (Fig. 5, D and E). Serum insulin also increased in DIO mice after 4 and 9 wk of treatment with 0.1 mg/kg GSK’157, although the differences were not statistically significant (Fig. 5 F).

The diverse set of data obtained with opposite pharmacological manipulations of the ISR revealed that ISR activation with HF ameliorates phenotypes in DIO mice whilst inhibition of the ISR with low, ISR-inhibiting doses of PERK inhibitor GSK’157 aggravated glucose intolerance.

### Harnessing GNC2 to activate the ISR and protect from DIO

Here, we show that increasing ISR signaling with HF, an orally available activator of GCN2 that has progressed to phase 2 clinical trials for various indications, reduced glucose intolerance, weight gain, insulin resistance, and serum insulin levels in DIO mice. Importantly, we also show that treatment of DIO mice with low, ISR-inhibiting doses of PERK inhibitor GSK’157 aggravates glucose intolerance. Thus, the work presented here provides a comprehensive foundation to support the concept of ISR activation for ameliorating diabetes phenotypes in DIO mice. HF has broad tissue distribution, and this might be advantageous as various tissues may contribute to the therapeutic benefits observed here.

Genetic and mechanistic studies over the past two decades have revealed that eIF2α phosphorylation by PERK is essential to maintain glucose homeostasis in mice and humans (Delépine et al., 2000). Here, we successfully harness the ISR for therapeutic benefit in a mouse model of DIO. In addition to the translational potential of this study, our findings provide important insights about the importance of the ISR in DIO.

The ISR has expanded considerably between yeast and mammals with yeast encoding for only one eIF2 kinase, GCN2, whilst mammals have evolved with PERK, HRI, and PKR (Sonenberg and Hinnebusch, 2009; Wek, 2018). GCN2 is activated by amino acid starvation and maintains amino acid homeostasis in yeast (Dever et al., 1992). In humans, loss of function mutations in GCN2 are viable but cause pulmonary veno-occlusive disease (PVOD), a rare form of pulmonary arterial hypertension (Montani et al., 2016). PVOD has both a genetic and an environmental component and develops in individuals lacking a functional GCN2 only upon exposure to drugs, toxins, or radiation (Montani et al., 2016). Thus, loss of GCN2 function in humans, in most cases, is not detrimental. In contrast, loss of function mutations in PERK are inevitably fatal and cause juvenile insulin-dependent diabetes (Delépine et al., 2000). This genetic information establishes that PERK is the essential ISR kinase for the maintenance of glucose homeostasis and β-cell function. The fact that loss of GCN2 function is not, on its own, pathogenic in humans implies that GCN2 has largely become dispensable in humans, whilst being the most ancient ISR kinase. This suggests that its activity might be minimal in humans probably because amino acid homeostasis is largely maintained through interorgan transport in mammals (Brosnan, 2003) rather than by cell-autonomous mechanism in unicellular yeast.

Here, we show that we can harness GCN2 kinase and activate it to treat DIO in mice. Whilst it has been genetically demonstrated in mice and humans that PERK is the primary kinase that evolved to maintain glucose homeostasis, we propose that its protective function reaches the limit of its capacity in HFD with excessively high glucose levels. Here, we show that we can harness the presumably dormant GCN2 with HF to pharmacologically increase the capacity and performance of the ISR in DIO mice. Thus, we propose that one can increase the benefit and adaptive capacity of the ISR for therapeutic purposes by activating one of its dispensable kinases. These findings have both conceptual and translational relevance.

## Materials and methods

### Study design

ISR has emerged as a prime target for new pharmacological manipulations with compounds that either prolong or inhibit the ISR (Luh and Bertolotti, 2020; Costa-Mattioli and Walter, 2020). To evaluate the therapeutic potential of harnessing the ISR in DOI, we treated mice with two compounds with opposite ISR activity: an ISR activator halofuginone (HF) and ISR inhibitor GSK’157. An important factor in this study design is the fact that HF is an orally available compound that has progressed to phase 2 clinical trials in humans, and GSK’157 has gone through late preclinical development.

First, we set out to assess the effectiveness of HF on in-house bred 6–7-wk old C57BL/6J male mice pancreas and found that a single oral dose (HF 0.50 mg/kg) significantly upregulated the expression of key ISR genes 4 h after dosing. We then generated DIO cohorts of inbred 6–7-wk old C57BL/6J male mice by feeding on HFD (60% kcal) for 21 wk. Development of diabetes-related symptoms was confirmed in these mice after 12 wk of HFD with impaired glucose tolerance, increased insulin resistance, and enhanced weight gain. These mice were then divided into three groups that were daily orally dosed with either placebo, HF (0.08 mg/kg), or HF (0.20 mg/kg) whilst continuing feeding on HFD. The efficacy of the treatment was monitored following weight gain, glucose tolerance, insulin resistance, serum insulin, and C-peptide levels. We then performed a similar study by daily oral dosing DIO mice with ISR inhibitor GSK’157. ISR activation with HF or inhibition with GSK’157 was assessed at the end stage following expression of the ISR markers in the pancreas after 9 wk of treatment. The number of mice used per experiment and statistical analysis used are indicated in figure legends. The HF study was replicated in a separate cohort to assess the robustness of the findings. All the experiments conducted were approved by the local animal welfare and ethical review board and followed the guidelines from the Home Office, UK. These data indicated the therapeutic effect of activating the ISR with HF to ameliorate phenotypes in DIO mice. This study is the first side-by-side comparison of antagonistic manipulations of the ISR, in a disease model, which can serve as a paradigm for other conditions.

### Ethical statement

All animal work undertaken in this study was done with the approval of the LMB Animal Welfare and Ethical Review Body (AWERB) and the UK Home Office, under project license number P9DCDB3BO.

### Animal housing and husbandry

In-house bred 6–7-wk old C57BL/6J male mice were used for this study. All experimental mice were housed and cared for in compliance with the Home Office Code of Practice for the Housing and Care of Animals used in Scientific Procedures. This study followed the ARRIVE guidelines (du Sert et al., 2020).

All animal experiments performed in this study were approved by the Home Office UK. All experimental animals were housed in specific pathogen-free ventilated cages (Tecniplast GM500; Tecniplast) on Eco-pure chips 8 bedding (ECO8; Datesand) and Enviro-Dri nesting material (034013; LBS) at 19–23°C with 12 h light-dark cycle with light from 7:00 AM to 7:00 PM. Animals were provided with an ad libitum supply of food and water along with enrichments including play tunnel (S-75 × 38.1 × 1.25 mm, CS3B01; Datesand), aspen bricks (small 5 × 1 × 1 cm, CS3C15; Datesand), and nesting material (034013; Enviro-Dri, LBS). Animals were fed with either a normal chow diet (Special diets services, 801722 CRM [P]) or a high-fat diet with 60% kcal fat (D12492i; Research diets). Mice were randomly allocated to treatment groups and experiments were performed in a blinded fashion. The maximum number of mice housed in an individual cage was 5. To monitor health, animals were visually checked every day and cleaned if soiled. Animals were weight monitored thrice weekly. Animals found sick (not due to drug treatment) or injured were excluded from the study.

### Weight monitoring

Mice were weight monitored three times per week for the entire period of study. Change in weight due to diet (HFD or ND) or drug treatment (Placebo or HF or GSK’157) was calculated by subtracting initial weight from newly recorded weight. Data were analyzed and represented using GraphPad prism.

### In vivo pharmacological treatments

For ISR induction, a commercially available oral (aqueous) solution of lactate salt of HF (Halocur 0.5 mg/ml; MSD Animal Health UK Limited) was used. For oral administration in mice, the stock solution (0.5 mg/ml) was appropriately diluted in sterile water, aliquoted, and stored at −20°C. Aliquots were thawed just before dosing. Experimental mice received either a single oral dose of 0.50 mg/kg or a single daily oral dose of 0.08 or 0.20 mg/kg of HF for 9 wk. Sterile water was used as a placebo. For ISR inhibition, GSK’157 (17372; Cayman Chemicals) was used. For oral administration, GSK’157 powder was dissolved in DMSO to prepare a stock solution of 10 mg/ml. The stock solution was aliquoted and stored at −20°C. Just before dosing, aliquots were thawed and appropriately diluted in sterile water. Mice were daily orally gavaged with 0.10 mg/kg of GSK’157 for 9 wk. For placebo treatment, 0.7–0.9% aqueous solution of DMSO was used. All the treatments were done in a blinded fashion.

### Glucose tolerance test

Mice were food-deprived for 13 h before receiving a single intraperitoneal injection of glucose (2 g/kg body weight) (G8270-1KG; Sigma-Aldrich). Blood glucose levels were measured at 0, 15, 30, 60, 90, and 120 min post glucose injection by collecting blood from the saphenous vein. AlphaTrak 2 Starter Kit, with an accuracy range of 20–750 mg/dl, was used to measure blood glucose levels by following the manufacturer’s instructions. 750 mg/dl was assigned for mice displaying blood glucose levels beyond the upper limit. Data analysis, representation, and calculation of the area under the curve were done using GraphPad prism. The area under the curve calculated individually for each experimental mouse was used together for statistical analysis.

### Insulin tolerance test

Mice were food-deprived for 5 h before receiving a single intraperitoneal injection of insulin (0.75 units/kg body weight) (VL7516, Humalog 100 U/ml; Eli Lilly). Blood glucose levels were measured at 0, 15, 30, and 60 min after insulin injection by collecting blood from the saphenous vein. AlphaTrak 2 Starter Kit, with an accuracy range of 20–750 mg/dl, was used to measure blood glucose levels by following the manufacturer’s instructions. Data analysis, representations, and calculations of the area under the curve were done using GraphPad prism. The area under the curve calculated individually for each experimental mouse was used together for statistical analysis.

### Food consumption

Food was weighed before putting in mice cages and after a week to calculate the total amount of food consumed per cage per week. Food consumed per mouse per week was calculated by dividing this value by the number of mice in the respective cages. Data analysis, representations, and calculations of the area under the curve were done using GraphPad Prism. The area under the curve calculated individually for each experimental mouse was used together for statistical analysis.

### Serum ELISA

Blood collected from mice was allowed to clot at room temperature for 30 min followed by spinning at 2,000 rcf for 10 min at room temperature. The supernatant (serum) was collected, snap-frozen in liquid nitrogen, and stored at −80°C. The serums were thawed on ice to be used in ELISA to measure insulin (Ultra-sensitive mouse insulin ELISA kit, 90080; Crystal Chem), glucagon (81518, New mouse glucagon ELISA kit; Crystal Chem), and C-peptide (90050, Mouse C-peptide ELISA kit; Crystal Chem) levels following manufacturer’s instructions. Where needed, serum samples were appropriately diluted in PBS to bring absorbance within the range of the calibration curve. For these samples, final concentration was calculated by multiplying with the dilution factor. Data from each week was analyzed separately and represented on a single plot. Data were analyzed and represented using GraphPad Prism.

### Quantitative real-time PCR

Mice were dissected to harvest the liver and pancreas. Pancreas were promptly harvested and immediately submerged in RNA protect solution (76106; Qiagen) to stabilize RNA by inactivating RNase present in the tissue, whereas livers were snap frozen in liquid nitrogen and stored at −80°C. Each pancreas was then transferred to 8 ml of ice-cold TRIzol reagent (15596018; Thermo Fisher Scientific) and homogenized, on ice using a handheld homogenizer (T 10 basic ULTRA-TURRAX, 0003737002; IKA), whereas ∼100 mg of frozen livers were homogenized in 1 ml of ice-cold TRIzol reagent (15596018; Thermo Fisher Scientific) using Precellys lysing kit (CK14, P000912-LYSK1-A.0; Bertin Technologies) and tissue homogenization system (Precellys 24; Bertin Technologies). Homogenates were centrifuged at 12,000 rcf for 5 min at 4°C to pellet undigested tissues. ∼0.5 ml supernatants were transferred to fresh 2 ml tubes with 100 µl of chloroform and vigorously shaken for ∼15 s. Mixtures were again centrifuged at 12,000 rcf for 15 min at 4°C followed by careful transfer of upper, RNA-containing, aqueous layer into fresh 2 ml tubes. To each tube, 1.5 volumes of isopropanol was added and mixed thoroughly. These mixtures were loaded in spin columns of RNeasy mini kit (17104; Qiagen), placed in 2 ml collection tubes, and centrifuged at 8,000 rcf for 15 s. The flowthroughs were discarded and spin columns were subjected to RNA extraction protocol of RNeasy mini kit from step 1–4 (part-2) followed by steps 5–8 (part-1), as described earlier (Azevedo-Pouly et al., 2014). After confirming the integrity of RNA via agarose gel electrophoresis, RNA concentrations were measured using NanoDrop 2000c Spectrophotometer (ND-2000C; Thermo Fisher Scientific). 500 ng RNA was used for cDNA synthesis using iScript cDNA synthesis kit (1708891; BioRad) following the manufacturer’s guidelines. The cDNAs were used for real-time qPCR on ViiA 7 Real-Time PCR system (Applied Biosystems) using SYBR select master mix (13256519; Applied biosystems) following manufacturer’s guidelines. Samples were run in triplicates, and RNA levels of β actin were used as internal control. To analyze fold change in expression for each gene, mean C_t_ values, from triplicate samples, were used. ∆C_t_ was calculated by normalizing the mean C_t_ values of each gene against the mean C_t_ values of β actin. The mean ∆C_t_ calculated for the placebo group was used to calculate ∆∆C_t_ for all genes. Fold change in expression was calculated using 2^−∆∆Ct^ formula. The following primers were used:

*Atf4*(F): 5′-GCCTG ACTCTGCTGCTTACA-3′, (R): 5′-TGGAGAAGGC AGATTGTC-3′,

*Asns*(F): 5′-ACA​CTC​CAC​CAC​TCC​CTT​CT-3′, (R): 5′-TGG​GAA​GAG​TTT​CTC​CAC​GC-3′,

*Chop*(F): 5′-AAC​AGA​GGT​CAC​ACG​CAC​AT-3′, (R): 5′-ACT​TTC​CGC​TCG​TTC​TCC​TG-3′, *Sesn2*(F): 5′-CCT​TCT​CCA​CAC​CCA​GAC​AT-3′, (R): 5′-AGC​CTC​TGG​ATC​AGC​GAG​TA-3′,

*Stc2*(F): 5′-TTC​GAT​GCC​CAG​GGA​AAG​TC-3′, (R): 5′-TCT​CCA​CAA​TCA​CAC​CGA​CG-3′, *Mthfd2*(F): 5′-CCG​CCA​GTC​ACT​CCT​ATG​TT-3′, (R): 5′-GGA​GGC​CAT​CTA​CGT​TCT​CA-3′,

β*-Actin*(F): 5′-CCA​GCC​TTC​CTT​CTT​GGG​TA-3′, (R): 5′-AGA​GGT​CTT​TAC​GGA​TGT​CAA​CG-3′. Data were analyzed and represented using GraphPad Prism.

### Cell culture

HeLa cells were cultured in Dulbecco’s Modified Eagle’s Medium (D5796; Thermo Fisher Scientific) added with glutamine (25030081; Thermo Fisher Scientific), 10% fetal bovine serum (10270106; Thermo Fisher Scientific), and penicillin-streptomycin mix (15140122; Thermo Fisher Scientific) and maintained in a humidified incubator with 5% CO_2_, at 37°C. AR42J cells (CRL-1492; ATCC) were cultured in Ham’s F-12K (Kaighn’s) Media (21127; Thermo Fisher Scientific) with 20% fetal bovine serum (10270106; Thermo Fisher Scientific) and penicillin-streptomycin mix (15140122; Thermo Fisher Scientific) and maintained in a humidified incubator with 5% CO_2_ at 37°C.

### Stock preparation of compounds for the treatment of cells

HF (Sigma-Aldrich, 32481), GSK’157 (17372; Cayman Chemicals), and Tunicamycin (T7765; Sigma-Aldrich) powders were dissolved in DMSO or water (Tunicamycin), aliquoted, stored at −20°C, and thawed just before use.

### Western blot

For HeLa cells, the day before treatment, 80,000 or 40,000 HeLa cells were seeded per well in 12- or 24-well plates, respectively. For AR42J cells, the day before treatment, 80,000 cells were seeded per well in 24-well plates. Cells were treated with increasing concentrations of halofuginone (5 h) or GSK’157 (2.5 h). For co-treatment with Tunicamycin, cells were first treated with GSK’157 for 30 min followed by the addition of Tunicamycin and incubation for a further 2 h. At the end of treatments, cells were washed with PBS and lysed in 150 µl (12-well plate) or 75 µl (24-well plate) Laemmli Buffer supplemented with DTT. Lysates were boiled at 95°C for 5 min, sonicated, and resolved on Bolt 4–12% Bis-Tris Plus (NW04120BOX; Thermo Fisher Scientific) or NuPAGE 3–8% Tris-Acetate gels (EA03755BOX; Thermo Fisher Scientific). Proteins were transferred to 0.2 µm nitrocellulose membranes using Trans-Blot Turbo Transfer Pack (1704158; Bio-Rad) and Trans-Blot Turbo Transfer System (1704150; Bio-Rad). Membranes were blocked in blocking buffer made with 5% skimmed milk in TBS-T (0.05% Tween) and then probed with primary antibodies appropriately diluted in 4% bovine serum albumin in TBS buffer (50 mM Tris-Cl at pH 7.5, 150 mM NaCl). Membranes were washed three times in TBS-T (0.05% Tween) buffer followed by incubation with appropriate horseradish peroxidase-conjugated secondary antibodies (anti-mouse W402B, 1:5,000, anti-rabbit W401B, 1:10,000; Promega) appropriately diluted in 4% bovine serum albumin in TBS buffer. Proteins were visualized using ECL Prime Western Blotting System (RPN2232; Cytiva) and imaged by ChemiDoc Touch system (Bio-Rad). The following primary antibodies were used: ATF4 (10835-1-AP, 1:1,000; Proteintech), α-tubulin (T5168, 1:5,000; Sigma-Aldrich), P-eIF2α (ab32157, 1:1,000; Abcam), eIF2α (L57A5, 1:1,000; Cell Signaling Technology), eIF2α (ab26197, 1:1,000; Abcam), GCN2 (3302S, 1:1,000; Cell Signaling Technology), P-GCN2 (ab75836, 1:1,000; Abcam), PERK (D11A8, 1:1,000; Cell Signaling Technology), Vinculin (4650 S, 1:1,000; Cell Signaling Technology), and β actin (ab6276, 1:5,000; Abcam).

### Statistics

Data for the glucose tolerance test and insulin tolerance test were analyzed via two-way ANOVA. Data for AUC of glucose tolerance test after treatment, AUC of insulin tolerance test, AUC of food consumption, serum ELISA for insulin, C-peptide and glucagon, and weight gain after treatment were analyzed by ordinary one-way ANOVA. Data for fold change in gene expression upon single oral dose of HF (0.5 mg/kg)/placebo and chronic treatment with GSK’157/placebo were analyzed by Mann–Whitney test whereas gene expression data for chronic treatment with placebo, HF (0.08 mg/kg), and HF (0.20 mg/kg) were analyzed by ordinary one-way ANOVA. Data of weight gain before treatment, AUC of glucose tolerance test before treatment and AUC of food consumption after GSK,157 treatment were analyzed by unpaired *t* test. Data is represented as mean ± SEM. Outlier data points were excluded from the analysis. n numbers are indicated in figure legends. GraphPad Prism (version 10.1.0) was used to analyze and represent data.

### Online supplemental material

Fig. S1 shows that oral administration of halofuginone improves glucose tolerance in DIO mice. Replicate of Fig. 2 with a separate DIO mouse cohort. Fig. S2 shows oral administration of halofuginone induces ISR in the liver of DIO mice. Fig. S3 shows that oral administration of halofuginone reduces insulin resistance and weight gain in DIO mice. Replicate of Fig. 3 with a separate DIO mouse cohort.

## Supplementary Material

SourceData F1 is the source file for Fig. 1.

SourceData F4 is the source file for Fig. 4.

## Data Availability

The data are available from the corresponding author upon reasonable request.

## References

[bib1] Atkins, C., Q. Liu, E. Minthorn, S.Y. Zhang, D.J. Figueroa, K. Moss, T.B. Stanley, B. Sanders, A. Goetz, N. Gaul, . 2013. Characterization of a novel PERK kinase inhibitor with antitumor and antiangiogenic activity. Cancer Res. 73:1993–2002. 10.1158/0008-5472.CAN-12-3109 23333938

[bib2] Azevedo-Pouly, A.C.P., O.A. Elgamal, and T.D. Schmittgen. 2014. RNA Isolation from mouse pancreas: A ribonuclease-rich tissue. J. Vis. Exp. e51779. 10.3791/51779 25145327 PMC4297470

[bib3] Bertolotti, A. 2018. The split protein phosphatase system. Biochem. J. 475:3707–3723. 10.1042/BCJ20170726 30523060 PMC6282683

[bib4] Brosnan, J.T. 2003. Interorgan amino acid transport and its regulation. J. Nutr. 133:2068S–2072S. 10.1093/jn/133.6.2068S 12771367

[bib5] Costa-Mattioli, M., and P. Walter. 2020. The integrated stress response: From mechanism to disease. Science. 368:eaat5314. 10.1126/science.aat5314 32327570 PMC8997189

[bib6] Daugschies, A., U. Gässlein, and M. Rommel. 1998. Comparative efficacy of anticoccidials under the conditions of commercial broiler production and in battery trials. Vet. Parasitol. 76:163–171. 10.1016/S0304-4017(97)00203-3 9615950

[bib7] Delépine, M., M. Nicolino, T. Barrett, M. Golamaully, G.M. Lathrop, and C. Julier. 2000. EIF2AK3, encoding translation initiation factor 2-α kinase 3, is mutated in patients with Wolcott-Rallison syndrome. Nat. Genet. 25:406–409. 10.1038/78085 10932183

[bib8] Dever, T.E., L. Feng, R.C. Wek, A.M. Cigan, T.F. Donahue, and A.G. Hinnebusch. 1992. Phosphorylation of initiation factor 2 alpha by protein kinase GCN2 mediates gene-specific translational control of GCN4 in yeast. Cell. 68:585–596. 10.1016/0092-8674(92)90193-g 1739968

[bib9] du Sert, N.P., V. Hurst, A. Ahluwalia, S. Alam, M.T. Avey, M. Baker, W.J. Browne, A. Clark, I.C. Cuthill, U. Dirnagl, . 2020. The ARRIVE guidelines 2.0: Updated guidelines for reporting animal research. PLoS Biol. 18:e3000410. 10.1371/journal.pbio.3000410 32663219 PMC7360023

[bib10] Harding, H.P., H. Zeng, Y. Zhang, R. Jungries, P. Chung, H. Plesken, D.D. Sabatini, and D. Ron. 2001. Diabetes mellitus and exocrine pancreatic dysfunction in perk-/- mice reveals a role for translational control in secretory cell survival. Mol. Cell. 7:1153–1163. 10.1016/S1097-2765(01)00264-7 11430819

[bib11] Keller, T.L., D. Zocco, M.S. Sundrud, M. Hendrick, M. Edenius, J. Yum, Y.J. Kim, H.K. Lee, J.F. Cortese, D.F. Wirth, . 2012. Halofuginone and other febrifugine derivatives inhibit prolyl-tRNA synthetase. Nat. Chem. Biol. 8:311–317. 10.1038/nchembio.790 22327401 PMC3281520

[bib12] Liu, M., Y. Huang, X. Xu, X. Li, M. Alam, A. Arunagiri, L. Haataja, L. Ding, S. Wang, P. Itkin-Ansari, . 2021. Normal and defective pathways in biogenesis and maintenance of the insulin storage pool. J. Clin. Invest. 131:e142240. 10.1172/JCI142240 33463547 PMC7810482

[bib13] Luh, L.M., and A. Bertolotti. 2020. Potential benefit of manipulating protein quality control systems in neurodegenerative diseases. Curr. Opin. Neurobiol. 61:125–132. 10.1016/j.conb.2020.02.009 32199101

[bib14] Misra, J., M.J. Holmes, E. T Mirek, M. Langevin, H.G. Kim, K.R. Carlson, M. Watford, X.C. Dong, T.G. Anthony, and R.C. Wek. 2021. Discordant regulation of eIF2 kinase GCN2 and mTORC1 during nutrient stress. Nucleic Acids Res. 49:5726–5742. 10.1093/nar/gkab362 34023907 PMC8191763

[bib15] Montani, D., E.M. Lau, P. Dorfmüller, B. Girerd, X. Jaïs, L. Savale, F. Perros, E. Nossent, G. Garcia, F. Parent, . 2016. Pulmonary veno-occlusive disease. Eur. Respir. J. 47:1518–1534. 10.1183/13993003.00026-2016 27009171

[bib16] Morikawa, S., and F. Urano. 2022. The role of ER stress in diabetes: Exploring pathological mechanisms using wolfram syndrome. Int. J. Mol. Sci. 24:230. 10.3390/ijms24010230 36613674 PMC9820298

[bib17] Pines, M., and I. Spector. 2015. Halofuginone—the multifaceted molecule. Molecules. 20:573–594. 10.3390/molecules20010573 25569515 PMC6272571

[bib18] Pitera, A.P., M. Szaruga, S.Y. Peak-Chew, S.W. Wingett, and A. Bertolotti. 2022. Cellular responses to halofuginone reveal a vulnerability of the GCN2 branch of the integrated stress response. EMBO J. 41:e109985. 10.15252/embj.2021109985 35466425 PMC9156968

[bib19] Rojas-Rivera, D., T. Delvaeye, R. Roelandt, W. Nerinckx, K. Augustyns, P. Vandenabeele, and M.J.M. Bertrand. 2017. When PERK inhibitors turn out to be new potent RIPK1 inhibitors: Critical issues on the specificity and use of GSK2606414 and GSK2656157. Cell Death Differ. 24:1100–1110. 10.1038/cdd.2017.58 28452996 PMC5442476

[bib20] Rozpędek-Kamińska, W., N. Siwecka, A. Wawrzynkiewicz, R. Wojtczak, D. Pytel, J.A. Diehl, and I. Majsterek. 2020. The PERK-dependent molecular mechanisms as a novel therapeutic target for neurodegenerative diseases. Int. J. Mol. Sci. 21:2108. 10.3390/ijms21062108 32204380 PMC7139310

[bib21] Scheuner, D., B. Song, E. McEwen, C. Liu, R. Laybutt, P. Gillespie, T. Saunders, S. Bonner-Weir, and R.J. Kaufman. 2001. Translational control is required for the unfolded protein response and in vivo glucose homeostasis. Mol. Cell. 7:1165–1176. 10.1016/S1097-2765(01)00265-9 11430820

[bib22] Sonenberg, N., and A.G. Hinnebusch. 2009. Regulation of translation initiation in eukaryotes: Mechanisms and biological targets. Cell. 136:731–745. 10.1016/j.cell.2009.01.042 19239892 PMC3610329

[bib23] Stecklair, K.P., D.R. Hamburger, M.J. Egorin, R.A. Parise, J.M. Covey, and J.L. Eiseman. 2001. Pharmacokinetics and tissue distribution of halofuginone (NSC 713205) in CD2F1 mice and Fischer 344 rats. Cancer Chemother. Pharmacol. 48:375–382. 10.1007/s002800100367 11761455

[bib24] Sundrud, M.S., R. Mazitschek, and M. Whitman. 2009. Halofuginone inhibits T17 cell differentiation by activating the amino acid starvation response. Science. 324:1334–1338. 10.1126/science.1172638 19498172 PMC2803727

[bib25] Szaruga, M., D.A. Janssen, C. de Miguel, G. Hodgson, A. Fatalska, A.P. Pitera, A. Andreeva, and A. Bertolotti. 2023. Activation of the integrated stress response by inhibitors of its kinases. Nat. Commun. 14:5535. 10.1038/s41467-023-40823-8 37684277 PMC10491595

[bib26] Tian, X., S. Zhang, L. Zhou, A.A. Seyhan, L. Hernandez Borrero, Y. Zhang, and W.S. El-Deiry. 2021. Targeting the integrated stress response in cancer therapy. Front. Pharmacol. 12:747837. 10.3389/fphar.2021.747837 34630117 PMC8498116

[bib27] Wek, R.C. 2018. Role of eIF2α kinases in translational control and adaptation to cellular stress. Cold Spring Harbor Perspect. Biol. 10:a032870. 10.1101/cshperspect.a032870 PMC6028073 29440070

