# Supplementary material for: Integrated stress response activator halofuginone protects mice from diabetes-like phenotypes
Source: J Cell Biol. 2024 Aug 16;223(10):e202405175. doi: 10.1083/jcb.202405175 (PMC11329777; doi:10.1083/jcb.202405175)
Supplement: SourceData F1 — is the source file for Fig. 1. [file JCB_202405175_SourceDataF1.pdf]

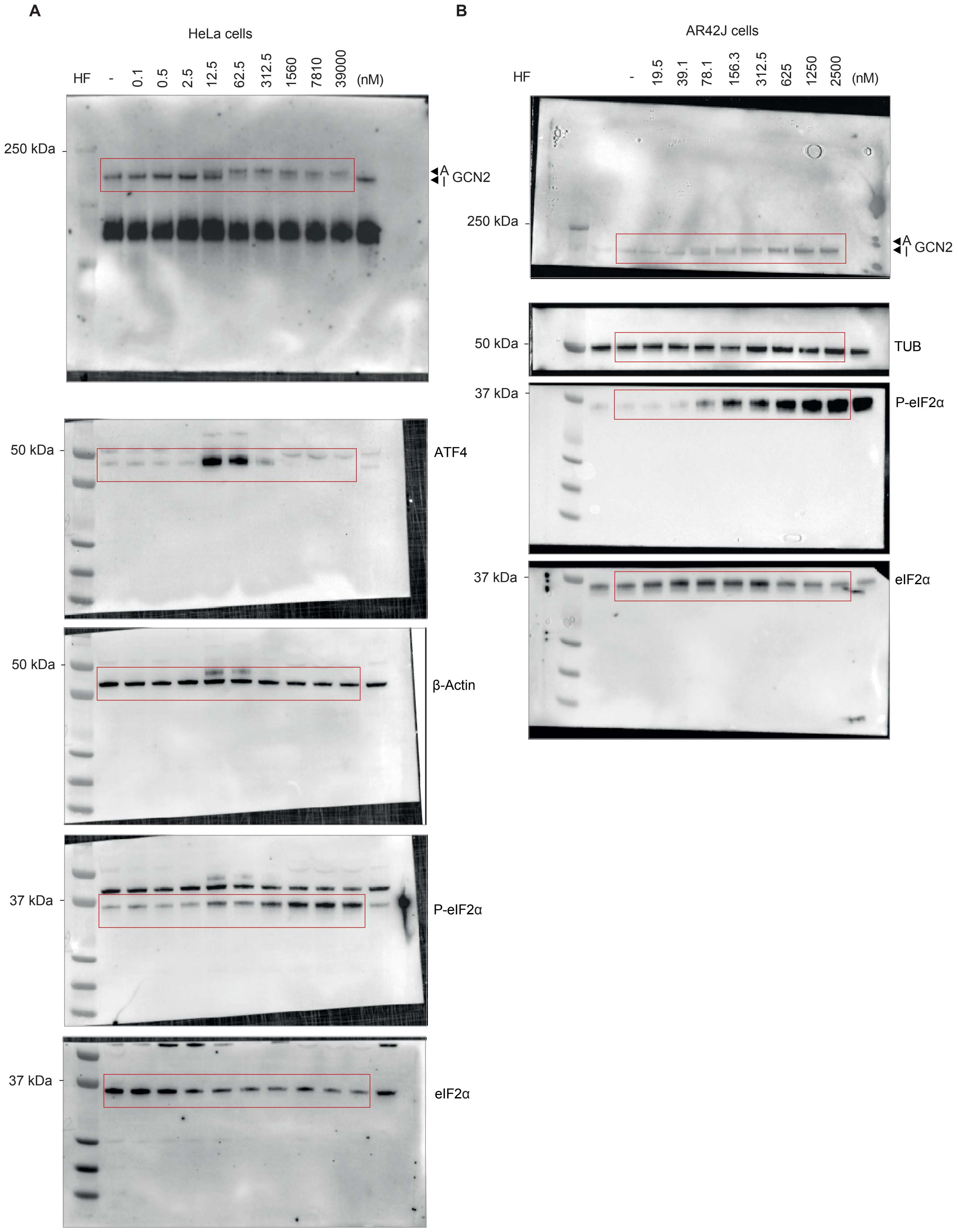
A
HeLa cells
HF - 0.1 0.5 2.5 12.5 62.5 312.5 1560 7810 39000 (nM)
250 kDa
A
I
GCN2
50 kDa
ATF4
50 kDa
β-Actin
37 kDa
P-eIF2α
37 kDa
eIF2α
B
AR42J cells
HF - 19.5 39.1 78.1 156.3 312.5 625 1250 2500 (nM)
250 kDa
A
I
GCN2
50 kDa
TUB
37 kDa
P-eIF2α
37 kDa
eIF2α
