# Supplementary figures and images for: Integrated stress response activator halofuginone protects mice from diabetes-like phenotypes
Source: J Cell Biol. 2024 Aug 16;223(10):e202405175. doi: 10.1083/jcb.202405175 (PMC11329777; doi:10.1083/jcb.202405175)

**A**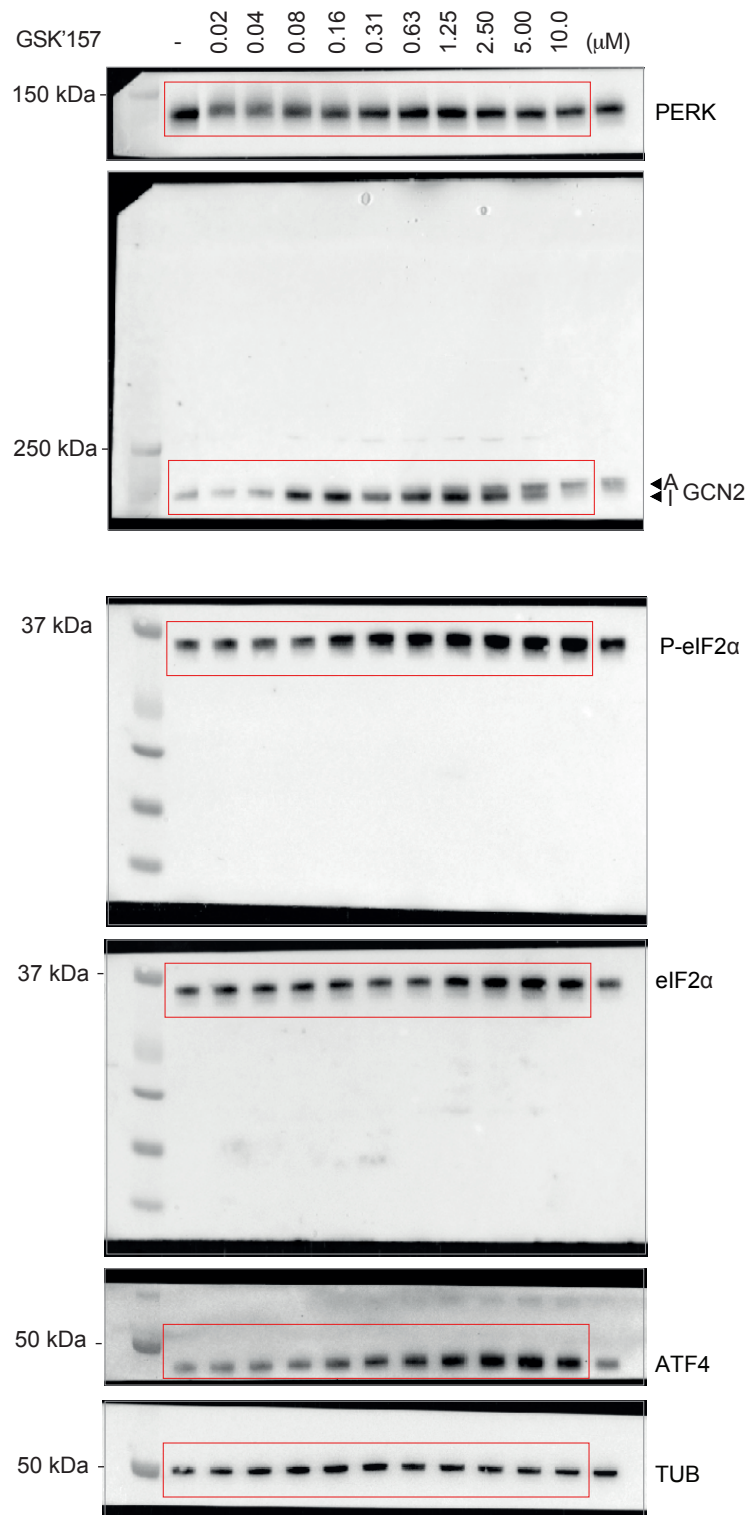**B**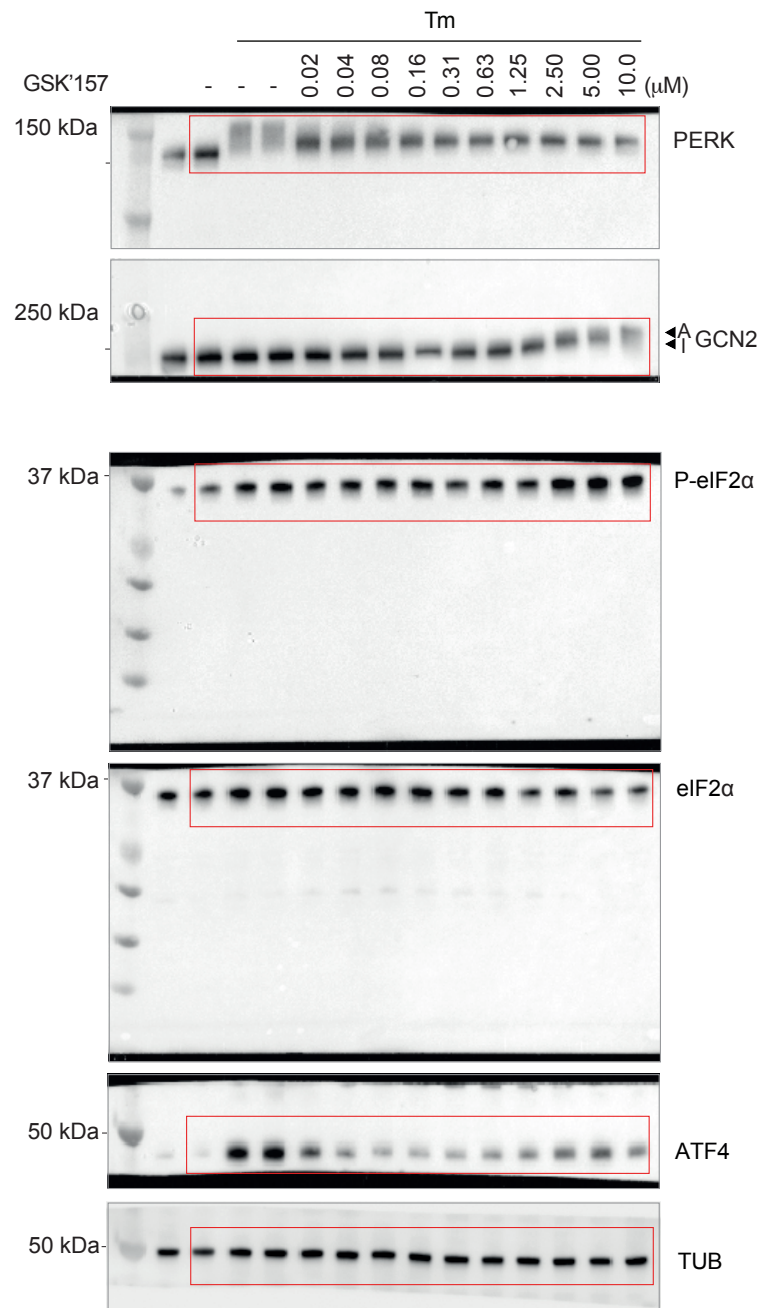

Supplement: SourceData F4 — is the source file for Fig. 4. [file JCB_202405175_SourceDataF4.pdf]
